# Association of vitamin D receptor gene polymorphism with the risk of sepsis: A systematic review and meta-analysis

**DOI:** 10.1097/MD.0000000000035130

**Published:** 2023-09-22

**Authors:** Qian Li, Wen Li, Menglu Chen, Yihui Chai, Liancheng Guan, Yunzhi Chen

**Affiliations:** a Guizhou University of Traditional Chinese Medicine, Guiyang City, Guizhou Province, China; b The Second Affiliated Hospital of Guizhou University of Traditional Chinese Medicine, Guiyang City, Guizhou Province, China.

**Keywords:** meta-analysis, polymorphism, sepsis, vitamin D receptor

## Abstract

**Background:**

To investigate the association between sepsis and the vitamin D receptor (VDR) gene polymorphisms.

**Methods:**

Databases including PubMed, Cochrane Library, EMbase, CNKI, Wanfang Data, and VIP Data were systematically searched. The association was assessed using odds ratios (ORs), and 95% confidence intervals (CIs). The statistical tests were performed using Review Manager 5.4.

**Results:**

We identified a total of 5 studies. The relationship between VDR gene polymorphisms (Apa I, Bsm I, Taq I, and Fok I), and incidence of sepsis was investigated. The results of this meta-analysis showed that the allelic contrast model (F vs f, *P* = .03, OR = 0.65, 95% CI = 0.44–0.95), dominant genetic model (FF vs Ff + ff, *P* = .02, OR = 0.53, 95% CI = 0.30–0.91), and codominance genetic model (FF vs ff, *P* = .03, OR = 0.39, 95% CI = 0.16–0.91) of VDR Fok I locus increased the risk of sepsis, and the lack of association between the VDR Fok I gene polymorphism and the risk assessment of sepsis, based on the ethnic subgroup analysis, might be attributable to the small sample size. The risk of sepsis with Apa I, Bsm I, and Taq I did not appear to be correlated.

**Conclusion subsections:**

This meta-analysis revealed that the VDR Fok I polymorphism is closely associated with the susceptibility to sepsis, and patients with sepsis have lower 25-hydroxyvitamin D levels. VDR Fok I gene mutations may change the risk of sepsis.

## 1. Introduction

Sepsis is a syndrome of physiological, pathological, and biochemical abnormalities caused by infection, which can lead to multiple organ dysfunction.^[1,2]^ Clinically, the common severe infectious diseases that frequently occur after burns, severe trauma, and surgery are characterized by severe illness, rapid progress, and high mortality.^[3,4]^ The incidence rate of sepsis is 3%, and the mortality can be as high as 30%.^[5,6]^ The pathogenesis of sepsis mainly progresses from inflammatory activation in the initial persistent inflammatory response to immune deficiency in the final immunosuppressive response, disrupting the body immune balance.^[7,8]^

Vitamin D_3_ (VD) is a natural form of VD produced in the skin by 7-dehydrocholesterol. Vitamin D receptor (VDR) is a steroid receptor that exerts biological effects on 1,25-dihydroxyvitamin D_3_ (1,25(OH)_2_D_3_).^[9,10]^ VDR gene is located at chromosomes 12q12 and q14.^[11]^ The metabolic process of VD is mainly completed in the liver and kidneys, and is hydroxylated by 25-hydroxylase (CYP27A1) to 25 hydroxyvitamin D_3_ (25(OH)D_3_), followed by the action of 1α-hydroxylase (CYP27B1), it becomes 1,25(OH)_2_D_3_. Among them, 1,25(OH)_2_D_3_ can bind to the VDR and produce dimers with the retinoid X receptor to bind to the target gene, regulating the transcription and expression of the target gene^[12]^; The criteria for determining the adequacy of VD are mainly based on the content of 25(OH)D_3_ in serum.^[13,14]^

There are several meta-analyses reporting the association of VDR gene polymorphisms and the risk of some diseases. For example, Mukhtar-Maryam et al^[15]^ conducted that VDR gene polymorphism can limit the anti-inflammatory effect of vitamin D by altering the 1,25(OH)_2_D_3_ binding site, thereby affecting the progression of arthritis in patients. Li Hui-Min et al^[16]^ indicated that VDR Bsm I and Taq I polymorphisms are associated with susceptibility to spinal osteoarthritis in a meta-analysis. Lv et al^[17]^ considered that the Fok I gene polymorphism may affect the risk, severity and cognitive function of Parkinson patients. The association of VDR polymorphisms and sepsis caused significant clinical and epidemiological research in recent years, but the reported results have been inconsistent. Moreover, the epigenome comprising different mechanisms, for example, DNA methylation, remodeling, histone tail modifications, chromatin microRNAs and long non-coding RNAs.^[18]^ Numerous lines of evidence suggest the influence of epigenome variation on health and production.^[19]^ The inforsmation obtained from the analysis of biological data by bioinformatics, in aligning sequences in information banks to find gene similarities and differences, predict the structure and function of gene products, diseases, and find phylogenetic relationships.^[20]^ It helps between genes and protein sequences.^[21]^ Therefore, we conducted a meta-analysis of the existing published studies on this topic to evaluate the strength of the association among the 4 main VDR gene polymorphisms (Apa I, Bsm I, Taq I, and Fok I) with the risk of sepsis.

## 2. Methods

### 2.1. Retrieval strategy

The Preferred Reporting Items for Systematic Reviews and Meta-Analysis (PRISMA) statement serves as a reporting guide for this systematic review (Table Supplemental 1, describing the PRISMA checklist, http://links.lww.com/MD/K27). The review is registered in advance on the International Registration System Review and Meta-Analysis Protocol Platform (INPLASY), with ID of INPLASY202340045 (Protocol Supplemental 1, describing the protocol registered in advance in INPLASY, http://links.lww.com/MD/K28).

### 2.2. Literature search

Our 2 authors (LQ and CML) conducted searches on Chinese and foreign language databases such as PubMed, EMbase, Cochrane Library, Chinese National Knowledge Infrastructure (CNKI), Wanfang Database, and VIP Database, covering the period from database establishment to March 2023. Search by combining different search methods with the subject words “vitamin D receptor” or “vitamin D receptor polymorphism” or “1,25-Dihydroxycholicalferrol receptor” or “cholecalciferol receptor” or “VDR gene” and “sepsis” or “bloodstream Infection” or “septicemia” or “pyemia.”

### 2.3. Literature screening

Two authors independently screened the literature according to a pre designed table and conducted cross check on the results of the included studies. For studies with disagreements that are difficult to determine whether to include, the inclusion was discussed or decided by the third author (L.W.).

### 2.4. Literature selection

#### 2.4.1. Inclusion criteria

The study must be a case control study; All qualified studies evaluating the relationship between VDR polymorphisms and sepsis risk are the main results; Using computable or extractable data from odds ratio (OR) and 95% confidence interval (CI) for research; Include the genotype or allele distribution of cases and healthy individuals with VDR polymorphism in the study; The statistical method is reliable, and the control group conforms to the Hardy–Weinberg equilibrium (HWE); repeated articles, commentaries, editorials, case reports, book chapters, reviews, and research with insufficient data are all excluded. The application of these standards ultimately leads to 5 studies that meets the conditions for meta-analysis.

#### 2.4.2. Exclusion criteria

Literature types include reviews, systematic evaluations, meta-analyses, case reports, animal studies, in vitro studies, and editorial articles; The control group was not healthy; The control group did not conform to HWE; There are no OR values in the literature or the OR value cannot be calculated through data; Low quality literature with incomplete data or unclear description.

### 2.5. Deviation risk assessment

The effectiveness of inclusion in the study was evaluated separately by 2 reviewers (LQ and CML). Case-control studies were evaluated using the Newcastle Ottawa scale (NOS), with scores ranging from 0 to 4, 5 to 7, and 8 to 10 representing low, medium, and high quality, respectively.^[22]^ Finally, for statistical analysis, we selected a case-control study with a NOS score of 6.

### 2.6. Data extraction

The following data was extracted by 2 authors from the included studies. The name of the lead author and the year of publication of the study; Country; the basic characteristics of the subjects, such as research category, sample size, average age or age range, genotype, total sample size; taking VDR Fok I as an example, the required data were extracted according to the allelic contrast model (F vs f), dominant genetic model (FF vs Ff + ff), recessive genetic model (FF + Ff vs ff), codominance genetic model (FF vs ff), and overdominance genetic model (FF + ff vs Ff).

### 2.7. Statistical analysis

Using Review Manager 5.4, all of the gathered data was examined and reviewed. Allelic contrast model, dominant genetic model, recessive genetic model, codominance genetic model, and overdominance genetic model were the 5 models we employed. Conduct a stratified study by dividing the study population into 3 groups depending on race: African, Asian, and European populations. Use each study OR and 95% CI to determine the size of the effects the research findings will have.

Utilizing the Q-test and *I*^2^ test, assess and analyze the heterogeneity of the contained literature. When *I*^2^ ≦ 50%, it shows no heterogeneity, and when *I*^2^ > 50%, it indicates heterogeneity. If the *P* > .1of the Q test, it is regarded that there is no heterogeneity; otherwise, it is thought that there is heterogeneity. A random effects model is utilized for the research if *P* < .1 and *I*^2^ > 50% indicate that there is considerable heterogeneity between the studies. Otherwise, the study used a fixed effects model. Articles related to evaluating the association between VDR gene polymorphisms and sepsis were consistent with the HWE. The funnel plot is investigated as potential publication bias.

## 3. Results

### 3.1. Study characteristics

**Figure 1.**
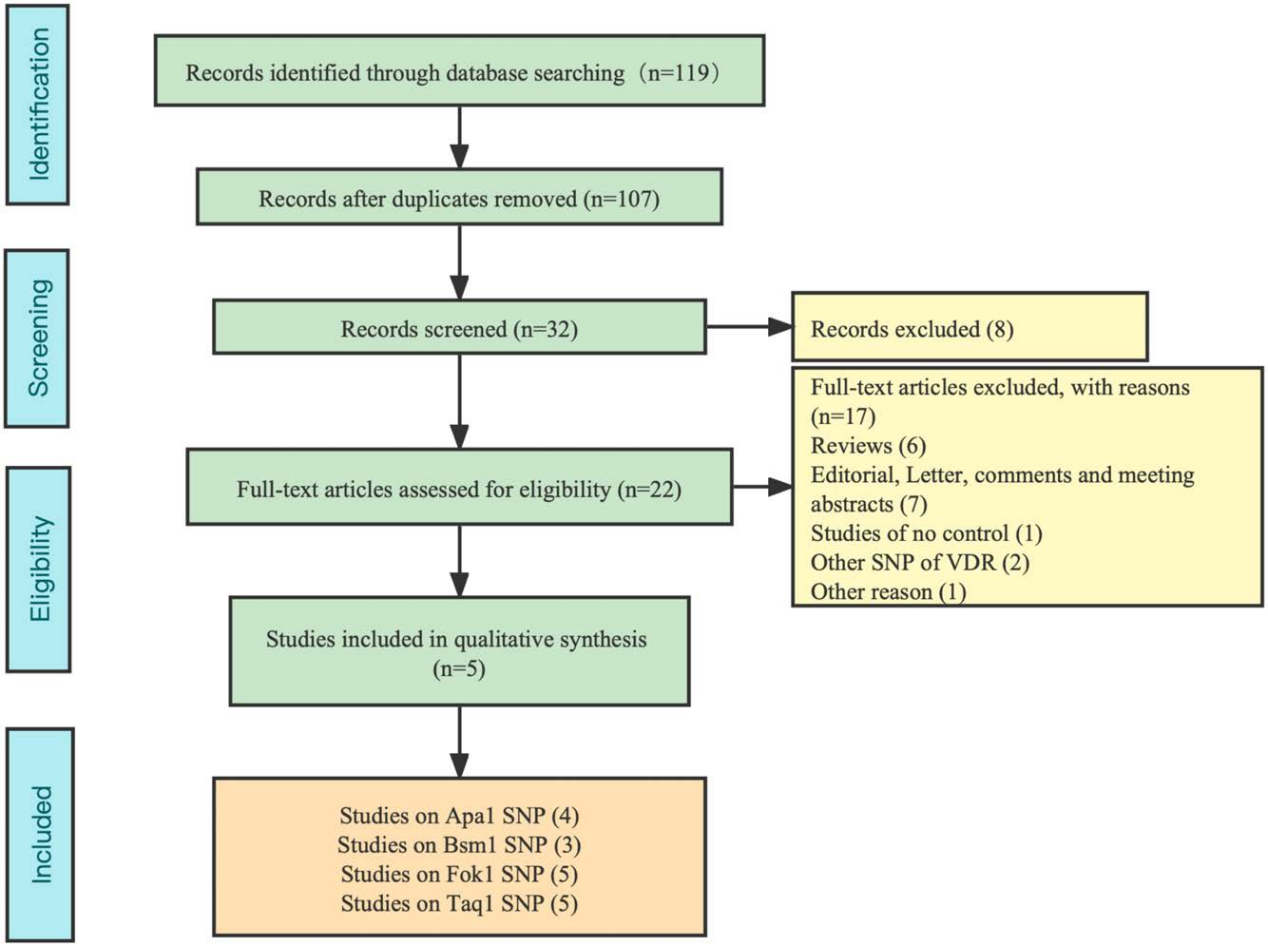
Flow diagram of the study identification.

A total of 119 articles were retrieved. After duplicates were removed, only 107 full-text studies were evaluated. Through further screening, 32 articles met the criteria, excluding 2 articles about other SNP of VDR^[23,24]^ and eventually 5 studies^[25–29]^ were included in the final meta-analysis for VDR gene polymorphisms and sepsis. In the study of VDR gene polymorphisms, the genotype frequencies of the control group were consistent with the HWE. The results of NOS showed that the quality of the methodology was generally good (Fig. 1). The fundamental features and allele frequency of the collected studies as shown in Table 1.

**Table 1:** Characteristics of studies reporting the distribution of VDR gene polymorphisms (Apa I, Bsm I, Fok I, and Taq I) in cases and controls.

Study	Yr	Ethnicity	Total		Allele frequency (case)				Genotyping methods	Age		NOS
			Case	Control	ApaI	BsmI	TaqI	FokI		Case	Control	
Tayel	2018	Africa	40	40	-	-	0.4	0.35	real time-PCR	38.3 ± 0.5 (wk)	38.5 ± 0.5 (wk)	6
Shaheen	2022	Africa	50	100	0.75	0.63	0.46	0.72	PCR-RFLP	37.54 ± 42.87 (mo)		6
Das	2016	Asia	60	60	0.417	0.43	0.533	0.517	PCR-RFLP	>37 (wk)		8
Yang	2022	Asia	576	421	0.62	0.875	0.79	0.71	real time-PCR	53.9 ± 12.7 (yr)	56.8 ± 15.8 (yr)	7
Zeljic	2017	Europe	100	104	0.42	-	0.625	0.395	real time-PCR	-	-	6

### 3.2. VDR gene polymorphism and the risk of sepsis

Distribution of Apa I, Bsm I, Taq I, and Fok I genotypes in patients with sepsis and controls as shown in Table 2.

**Table 2:** Distribution of genotype among sepsis patients and controls.

First author	Yr	Sepsis			Control		
ApaI (rs7975232)		AA	Aa	aa	AA	Aa	aa
Shaheen	2022	31	13	6	54	40	6
Das	2016	8	34	18	16	30	14
Yang	2022	242	230	104	168	178	75
Zeljic	2017	20	44	36	24	36	44
BsmI (rs1544410)		BB	Bb	bb	BB	Bb	bb
Das	2016	23	17	10	48	38	14
Yang	2022	2	48	10	22	36	2
Zeljic	2017	455	98	23	341	67	13
TaqI (rs731236)		TT	Tt	tt	TT	Tt	tt
Tayel	2018	6	20	14	13	16	11
Shaheen	2022	14	18	18	26	50	24
Das	2016	7	50	3	14	40	6
Yang	2022	357	196	23	269	143	9
Zeljic	2017	36	53	11	49	41	14
FokI (rs2228570)		FF	Ff	ff	FF	Ff	ff
Tayel	2018	5	18	17	13	21	6
Shaheen	2022	27	18	5	44	44	12
Das	2016	6	50	4	12	40	8
Yang	2022	288	242	46	274	130	17
Zeljic	2017	13	53	34	36	55	13

#### 3.2.1. Association of Apa I with sepsis

VDR Apa I polymorphism and its association with sepsis, there was no obvious heterogeneity in the allelic contrast model (*I*^2^ = 0%, *P* = .44), the dominant genetic model (*I*^2^ = 37%, *P* = .19), and the recessive genetic model (*I*^2^ = 0%, *P* = .41), so the Mantel-Haenszel fixed effects model was applied. No statistical association between VDR Apa I polymorphism and sepsis susceptibility were observed under the allelic contrast model (A vs a, *P* = .95, OR = 1.01, 95% CI = 0.86–1.17), dominant genetic model (AA vs Aa + aa, *P* = .80, OR = 1.03, 95% CI = 0.83–1.28), recessive genetic model (AA + Aa vs aa, *P* = .49, OR = 0.90, 95% CI = 0.67–1.21). The above data and codominance genetic model, overdominance genetic model data are organized in Table 3.

**Table 3:** Main results of pooled ORs in meta-analysis of VDR gene polymorphisms.

SNP	Genetic model	Test of association			Test of heterogenicity	
		OR	95% CI	*P* value	*P* value	*I*^2^ (%)
ApaI	A vs a	1.01	0.86–1.17	0.95	0.44	0
	AA vs Aa + aa	1.03	0.83–1.28	0.80	0.19	37
	AA + Aa vs aa	0.90	0.67–1.21	0.49	0.41	0
	AA vs aa	0.93	0.69–1.25	0.62	0.33	13
	AA + aa vs Aa	1.04	0.84–1.29	0.70	0.13	47
BsmI	B vs b	0.67	0.42–1.09	0.11	0.02	74
	BB vs Bb + bb	0.49	0.18–1.36	0.17	0.002	84
	BB + Bb vs bb	0.57	0.29–1.12	0.10	0.23	33
	BB vs bb	0.30	0.07–1.35	0.12	0.004	82
	BB + bb vs Bb	0.79	0.46–1.37	0.41	0.08	59
TaqI	T vs t	0.85	0.72–1.00	0.05	0.85	0
	TT vs Tt + tt	0.82	0.66–1.02	0.07	0.21	32
	TT + Tt vs tt	0.76	0.52–1.11	0.16	0.30	18
	TT vs tt	0.64	0.42–1.00	0.05	0.72	0
	TT + tt vs Tt	0.81	0.53–1.25	0.35	0.03	62
FokI	**F vs f**	**0.65**	**0.44–0.95**	**0.03**	**0.002**	**76**
	**FF vs Ff + ff**	**0.53**	**0.30–0.91**	**0.02**	**0.01**	**69**
	FF + Ff vs ff	0.55	0.28–1.11	0.10	0.02	67
	**FF vs ff**	**0.39**	**0.16–0.91**	**0.03**	**0.008**	**71**
	FF + ff vs Ff	0.83	0.55–1.26	0.39	0.04	59

The bold values represents a meaningful comparison of different genetic model in SNP of Fokl.

#### 3.2.2. Association of Bsm I with sepsis

As for VDR Bsm I polymorphism and its relationship to sepsis, no significant heterogeneity was found under the allelic contrast model (*I*^2^ = 74%, *P* = .02), the dominant genetic model (*I*^2^ = 84%, *P* = .002) with heterogeneity, so the random effects model was applied. The recessive genetic model (*I*^2^ = 33%, *P* = .23), so the fixed effects model was applied. No statistical association between VDR Bsm I polymorphism and sepsis susceptibility were observed under the allelic contrast model (B vs b, *P* = .11, OR = 0.67, 95% CI = 0.42–1.09), dominant genetic model (BB vs Bb + bb, *P* = .17, OR = 0.49, 95% CI = 0.18–1.36). The risk of sepsis was linked to the overall results of the recessive genetic model (BB + Bb vs bb, *P* = .10, OR = 0.57, 95% CI = 0.29–1.12). But the Bsm I genotype with the risk of sepsis was not strongly associated to race since the performance differences between the groups were not significant following the examination of ethnic groupings (Table 3).

#### 3.2.3. Association of Taq I with sepsis

For VDR Taq I polymorphism and the risk of sepsis, no significant heterogeneity was found among the allelic contrast model (*I*^2^ = 0%, *P* = .85), the dominant genetic model (*I*^2^ = 32%, *P* = .21), and the recessive genetic model (*I*^2^ = 18%, *P* = .30), so the fixed effects model was applied. No statistical association between VDR Taq I polymorphism and sepsis susceptibility were observed under the allelic contrast model (T vs t, *P* = .05, OR = 0.85, 95% CI = 0.72–1.00), dominant genetic model (TT vs Tt + tt, *P* = .07, OR = 0.82, 95% CI = 0.66–1.02), recessive genetic model (TT + Tt vs tt, *P* = .16, OR = 0.76, 95% CI = 0.52–1.11) (Table 3).

#### 3.2.4. Association of Fok I with sepsis

**Figure 2.**
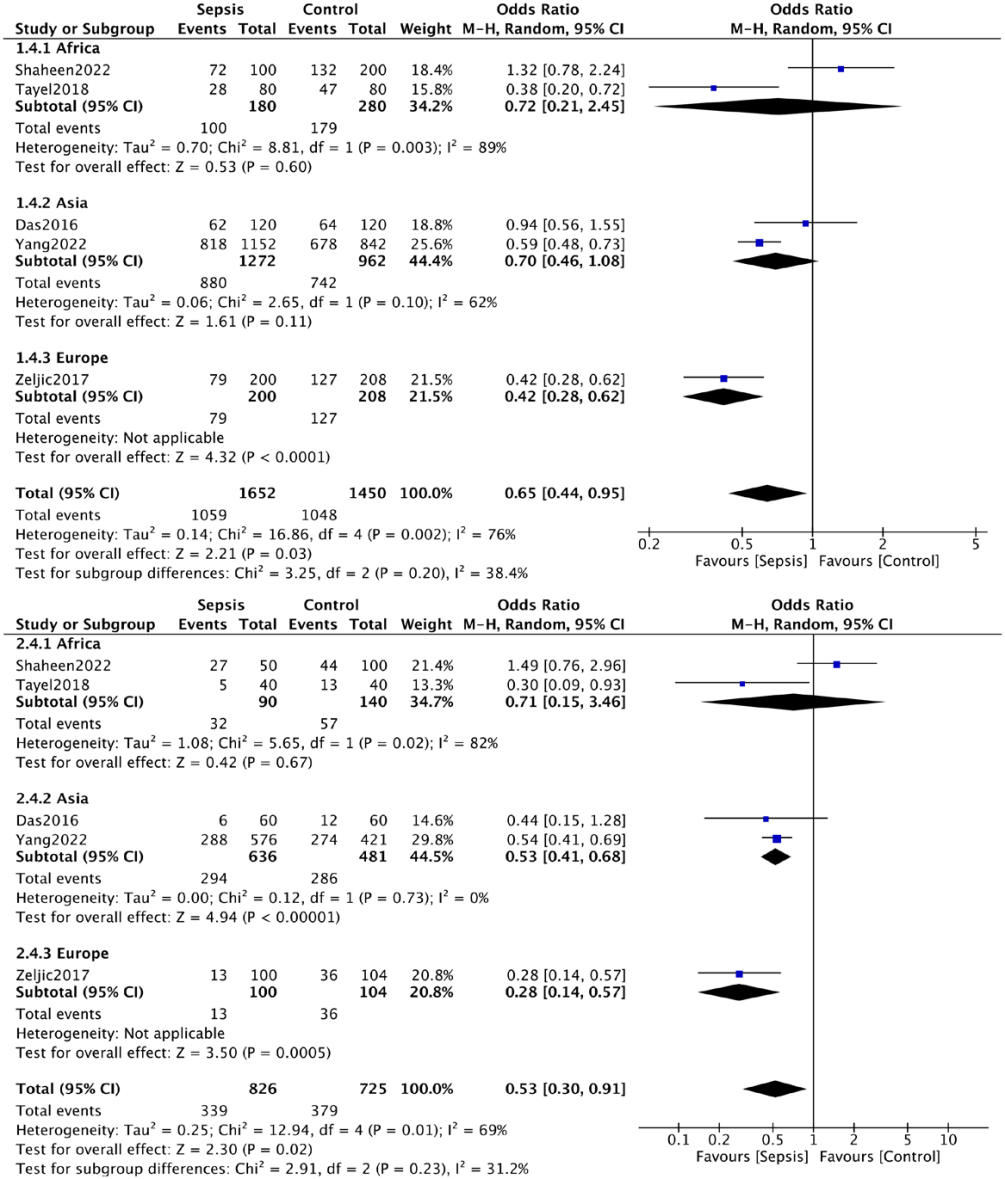
Forest plot of the association between the VDR Fok I polymorphisms and sepsis under the allelic contrast model (F vs f), dominant genetic model (FF vs Ff + ff) in different ethnicity subgroups. The horizontal lines correspond to the study-specific OR and 95% CI, respectively. The area of the squares reflects the study-specific weight. The diamond represents the pooled results of the OR and the 95% CI. CI = confidence interval, OR = odds ratio.

For VDR Fok I polymorphism and the risk of sepsis, 5 studies were included. The statistically heterogeneity was found under the allelic contrast model (*I*^2^ = 76%, *P* = .002), the dominant genetic model (*I*^2^ = 69%, *P* = .01), and the recessive genetic model (*I*^2^ = 67%, *P* = .02), so the random effects model was applied. The association between VDR Fok I polymorphism and sepsis susceptibility were observed under the allelic contrast model (F vs f, *P* = .03, OR = 0.65, 95% CI = 0.44–0.95), dominant genetic model (FF vs Ff + ff, *P* = .02, OR = 0.53, 95% CI = 0.30–0.91), recessive genetic model (FF + Ff vs ff, *P* = .10, OR = 0.55, 95% CI = 0.28–1.11) codominance genetic model (FF vs ff, *P* = .03, OR = 0.39, 95% CI = 0.16–0.91). We considered the forest plots of the allelic contrast model, dominant genetic model, and codominance genetic model as the representative (Fig. 2).

**Figure 3.**
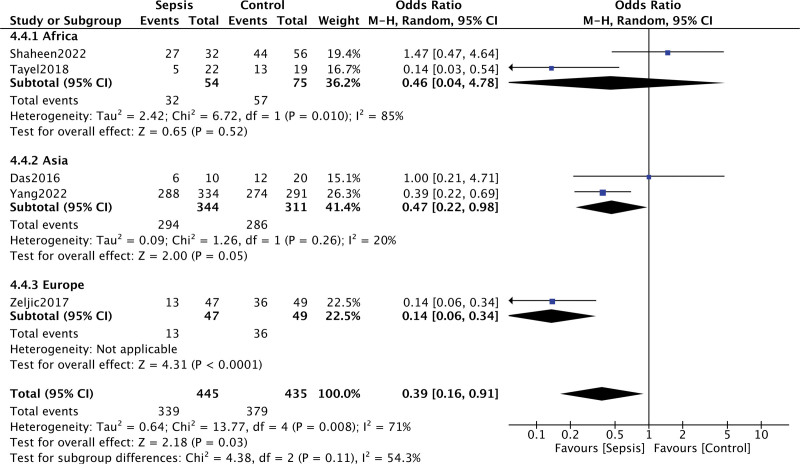
Forest plot of the association between the VDR Fok I polymorphisms and sepsis under the codominance genetic model (FF vs ff) in different ethnicity subgroups. The horizontal lines correspond to the study-specific OR and 95% CI, respectively. The area of the squares reflects the study-specific weight. The diamond represents the pooled results of the OR and the 95% CI. CI = confidence interval.

Under the codominance gene (FF vs ff, *P* = .03, OR = 0.39, 95% CI = 0.16–0.91) and overdominance gene (FF + ff vs Ff, *P* = .39, OR = 0.83, 95% CI = 0.55–1.26) of Fok I, heterogeneity was discovered. The link between VDR Fok I polymorphism and sepsis susceptibility were discovered via the codominance gene model (*I*^2^ = 71%, *P* = .008) and overdominance gene model (*I*^2^ = 59%, *P* = .04) after the random effect model was chosen. However, there was no statistically significant difference between the groups in the subgroup analysis. The effect of the Fok I gene polymorphism on sepsis is thought to be statistically significant rather than strongly tied to race, which may be due to the limited sample size (Fig. 3).

### 3.3. Publication bias

**Figure 4.**
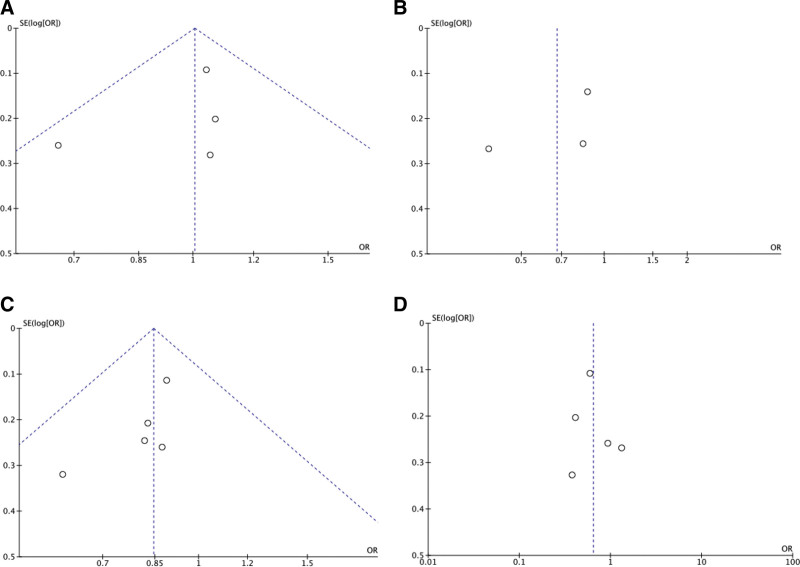
Funnel plot for the publication bias tests. Each point represents a separate study for the indicated association. The horizontal and vertical axis correspond to the OR and CIs. (A) VDR Apa I polymorphism and sepsis under the allelic contrast model (A vs a); (B) VDR Bsm I polymorphism and sepsis under the allelic contrast model (B vs b); (C) VDR Taq I polymorphism and sepsis under the allelic contrast model (T vs t). (D) VDR Fok I polymorphism and sepsis under the allelic contrast model (F vs f). CI = confidence interval, OR = odds ratio.

The publication bias of the individual studies was evaluated using funnel plots. We considered the figure of the allelic contrast model of Apa I, Bsm I, Taq I, and Fok I polymorphisms as representative. No visual publication bias was found in the funnel plot for the Apa I, Bsm I, Taq I, or Fok I polymorphisms using allelic contrast, as shown in Figure 4.

### 3.4. 25(OH)D_3_ level in sepsis

**Figure 5.**
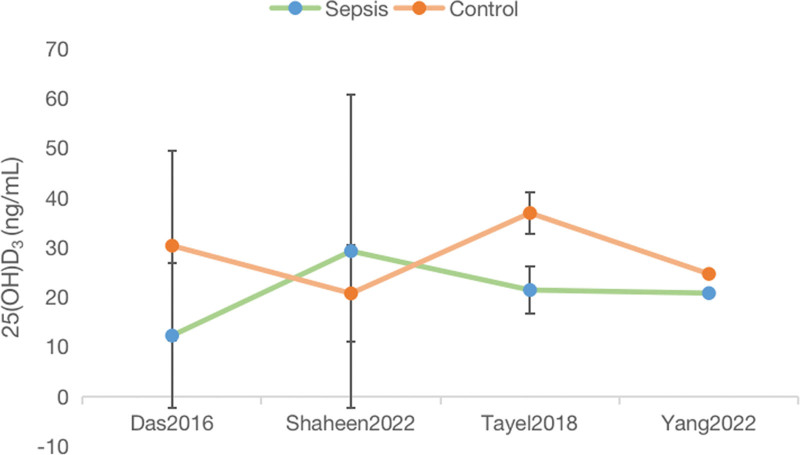
The level of Vitamin D in included studies. Note: Vitamin D levels were categorized as sufficient (>30 ng/mL), insufficient (>10 and < 30 ng/mL) or deficient (<10 ng/mL).

Following a trend description of the 25(OH)D_3_ level (Mean ± SD) among the 5 included articles (Fig. 5), Zeljic^[25]^ without pertinent data was eliminated. In the 4 articles, 3 articles of the research remaining investigations supported the finding that lower VD levels are expressed in sepsis patients, which was supported by the findings of related research.^[30,31]^ Vitamin D levels were categorized as sufficient (>30 ng/mL), insufficient (>10 and <30 ng/mL) or deficient (<10 ng/mL) on the basis of available references.^[32]^

## 4. Discussion

Vitamin D, as a lipid soluble hormone, can play an anti-inflammatory and regulatory role in the immune system. VDR plays an important role in inflammatory response, immune regulation, calcium and phosphorus metabolism, bone metabolism, oxidative stress, and other aspects.^[33]^ 1,25(OH)_2_D_3_ is the active form of vitamin D, mediating the biological effects of VDR on immune diseases. Recent studies have also shown that VDR polymorphisms play a major role in immune and inflammation status.^[34]^ Sepsis, as a systemic inflammatory response syndrome, is closely related to vitamin D. The multiple functions of vitamin D in the immune system ‘s response to infection may make it an important part of the fight against sepsis.^[31]^

The cause of sepsis is systemic organ dysfunction caused by the imbalance of immune response after the host is infected by pathogens, mainly manifested as enhanced systemic inflammatory response.^[34,35]^ Immune dysfunction, mitochondrial dysfunction, coagulation issues, neuroendocrine-immune network issues, endoplasmic reticulum stress, and autophagy are all factors in the pathophysiology of sepsis.^[36]^ Nowadays, sepsis is one of the leading causes of morbidity and mortality in intensive care units worldwide.^[37]^ Before appropriate antibiotic treatment, the survival rate of its severe form decreases by as much as 8 % per hour.^[38,39]^ There are no specific drugs for the treatment of sepsis, mainly focusing on anti-Toll-like receptor signaling pathway drugs, anti-endotoxin drugs, anti-cytokine drugs and high-dose glucocorticoid therapy.^[40]^ VD levels are associated with the risk of sepsis. Jeng^[41]^ evaluated that 100 % of patients with severe sepsis, 92 % of patients without sepsis and 66.5 % of healthy controls had VD deficiency. VDR has been found in most types of immune cells, including activated CD4 and CD8 T cells, B cells, neutrophils, macrophages and dendritic cells. VDR level can be used as an early warning for patients with sepsis.^[42,43]^ Therefore, VDR gene polymorphism may be closely related to the occurrence and development of sepsis. This meta-analysis can provide a direction for the study of VDR gene polymorphism and sepsis, and provide a favorable research perspective on the prevention and treatment of sepsis from the perspective of gene polymorphism.

There are He et al^[23]^ and Xiao et al^[24]^ also studied the risk analysis of VDR gene polymorphism and sepsis, but the VDR gene loci (rs739837, rs9729, rs2107301, rs2189480, rs2239185, rs3782905, rs4516035, rs7139166, rs11168266, and rs11168293) studied in these 2 articles do not conform to the Apa I (rs7975232), Bsm I (rs1544410), Taq I (rs731236), or Fok I (rs2228570) gene loci, and 2 articles have insufficient sample size, so they are excluded. We therefore conducted this meta-analysis, which included 5 studies on VDR gene polymorphisms. The allelic contrast model, dominant genetic model, codominance genetic model, and overdominance genetic model of Fok I in the VDR gene were shown to be associated with an increased risk of sepsis; this polymorphism may be a potential biomarker for early detection of sepsis. VDR is an effector receptor for vitamin D to activate transcription signal factors. Its Fok I polymorphism is associated with the risk of sepsis, and carrying allele F is a protective factor for sepsis, consistent with the results of existing research.^[25,28,29]^ Although there was no significant difference between ethnic groups after subgroup analysis of Fok I genotypes, the association between the risk of sepsis and the polymorphism of Fok I gene must exist. For Apa I, Bsm I, and Taq I polymorphism and the risk of sepsis, no significant heterogeneity was found among the dominant genetic model, recessive genetic model, and allelic contrast model. Above all, there are few studies on the effect of VDR gene polymorphism with the risk of sepsis, and we require more research.

The efficacy of statistical analysis may be impacted by the possible issue of heterogeneity. We made an effort to divide people into ethnic groupings, however this was unable to account for the disparity in results between research. Different experimental designs, diverse sample sizes, ethnic and geographic variances in the research population, gender and age inequalities in the participants, and various genotyping techniques all contribute to heterogeneity. Other limitations of this meta-analysis should also be acknowledged. First of all, only full-text publications in English and Chinese were made available. As a result, this meta-analysis did not include certain eligible papers that were unpublished or reported in other languages. Secondly, compared with Fok I and Taq I, there are fewer studies on ApaI and BsmI, and insufficient sample size may affect the results. Thirdly, some study studies lack data on gender or age, and other subgroup analyses have flaws that may have an impact on the variance in results. Overall, sepsis is affected by a variety of factors, including genetics,^[38]^ age,^[44,45]^ gender, and variances in sepsis,^[46]^ but the data currently available prevents us from performing subgroup analyses for each kind, necessitating more study.

Notwithstanding the study limitations, our meta-analysis has successfully and preliminary assessed the relationship between sepsis and the VDR gene polymorphism. Above all, the quality of the case-control studies was acceptable for producing worthwhile results. After that, the sample size will be increased to account for the shortcomings of the limited sample size of the research population. Finally, our work has avoided the errors made in earlier research, addressed them, and provided a reference for future research on sepsis and the VDR gene polymorphism.

## 5. Conclusions

In conclusion, this meta-analysis concludes that patients had a higher risk of sepsis due to the VDR Fok I polymorphism locus. However, this conclusion should be taken with care owing to the sample size restrictions. Therefore, to corroborate our findings, more studies with a bigger sample size are required, taking into account the interactions between genes and the environment. More clinical high-quality research in these regions should be carried out in the future to further confirm our findings. Prospective studies are also required to ascertain whether VDR can change the prognosis of sepsis.

## Acknowledgments

We thank Menglu Chen for article revision/review; Wen Li and Liancheng Guan for assistance in data curation; and Yihui Chai for help with visualization.

## Author contributions

**Conceptualization:** Qian Li.

**Data curation:** Qian Li, Wen Li, Liancheng Guan.

**Funding acquisition:** Yunzhi Chen.

**Project administration:** Yunzhi Chen.

**Visualization:** Qian Li, Yihui Chai.

**Writing – original draft:** Qian Li.

**Writing – review & editing:** Wen Li, Menglu Chen.

## Supplementary Material




