# Supplementary material for: Association of vitamin D receptor gene polymorphism with the risk of sepsis: A systematic review and meta-analysis
Source: Medicine (Baltimore). 2023 Sep 22;102(38):e35130. doi: 10.1097/MD.0000000000035130 (PMC10519506; doi:10.1097/MD.0000000000035130)
Supplement: Supplementary file 2 [file medi-102-e35130-s002.pdf]

International Platform of Registered Systematic Review and Meta-analysis Protocols

INPLASY PROTOCOL

To cite: Li et al. Association of vitamin D receptor gene polymorphism with the risk of sepsis: a meta-analysis. Inplasy protocol 202340045. doi: 10.37766/inplasy2023.4.0045

Received: 14 April 2023

Published: 14 April 2023

Corresponding author:
Yunzhi Chen

cyz2065@163.com

Author Affiliation:
Guizhou University of Traditional Chinese Medicine.

Support:
NSFC(82260890);2022NSRP of GZED(QTT [2022]023); 2022GGEIP(YCXZRB202209).

Review Stage at time of this submission: **Data analysis.**

Conflicts of interest:
None declared.

Association of vitamin D receptor gene polymorphism with the risk of sepsis: a meta-analysis

Li, Q[1]; Chen, ML[2]; Li, W[3]; Chai, YH[4]; Guan, LC[5]; Chen, YZ[6].

Review question / Objective: VDR gene polymorphism may be closely related to the occurrence and development of sepsis. This meta-analysis can provide a direction for the study of VDR gene polymorphism and sepsis, and provide a favorable research perspective on the prevention and treatment of sepsis from the perspective of gene polymorphism.To explore the relationship among the vitamin D receptor (VDR) gene polymorphisms and sepsis.

Eligibility criteria: Inclusion criteria: The study must be a case control study; All qualified studies evaluating the relationship between VDR polymorphisms and sepsis risk are the main results; Using computable or extractable data from odds ratio (OR) and 95% confidence interval (CI) for research; Include the genotype or allele distribution of cases and healthy individuals with VDR polymorphism in the study; The statistical method is reliable, and the control group conforms to the Hardy Weinberg equilibrium (HWE); Repeated articles, commentaries, editorials, case reports, book chapters, reviews, and research with insufficient data are all excluded. The application of these standards ultimately leads to 5 studies that meets the conditions for meta-analysis.

INPLASY registration number: This protocol was registered with the International Platform of Registered Systematic Review and Meta-Analysis Protocols (INPLASY) on 14 April 2023 and was last updated on 14 April 2023 (registration number INPLASY202340045).

INTRODUCTION

Review question / Objective: VDR gene polymorphism may be closely related to the occurrence and development of sepsis. This meta-analysis can provide a direction for the study of VDR gene polymorphism and sepsis, and provide a favorable research perspective on the prevention and treatment of sepsis from the perspective of gene polymorphism.To explore the relationship among the vitamin D receptor (VDR) gene polymorphisms and sepsis.

Li et al. Inplasy protocol 202340045. doi:10.37766/inplasy2023.4.0045 Downloaded from https://inplasy.com/inplasy-2023-4-0045/

Rationale: The association between VDR ApaI, BsmI, TaqI, and FokI gene polymorphism and sepsis patients has not been established. This meta-analysis hopes to establish a relationship between VDR ApaI, BsmI, TaqI, and FokI gene polymorphism and sepsis risk analysis.

Condition being studied: Sepsis is a syndrome of physiological, pathological, and biochemical abnormalities caused by infection, which can lead to multiple organ dysfunction. Clinically, the common severe infectious diseases that frequently occur after burns, severe trauma, and surgery are characterized by severe illness, rapid progress, and high mortality.

METHODS

Search strategy: Our two authors (LQ and CML) conducted searches on Chinese and foreign language databases such as PubMed, EMbase, Cochrane Library, Chinese National Knowledge Infrastructure (CNKI), Wanfang Database, and VIP Database, covering the period from database establishment to March 2023. Search by combining different search methods with the subject words "vitamin D receptor" or "vitamin D receptor polymorphism" or "1,25-Dihydroxycholicalferrol receptor" or "cholecalciferol receptor" or "VDR gene" and "sepsis" or "bloodstream Infection" or "septicemia" or "pyemia".

Participant or population: Patients with sepsis.

Intervention: None.

Comparator: Healthy Control.

Study designs to be included: Published case-control studies.

Eligibility criteria: Inclusion criteria: The study must be a case control study; All qualified studies evaluating the relationship between VDR polymorphisms and sepsis risk are the main results; Using computable or extractable data from odds ratio (OR) and 95% confidence interval (CI) for research; Include the genotype or allele distribution of cases and healthy individuals with VDR polymorphism in the study; The statistical method is reliable, and the control group conforms to the Hardy Weinberg equilibrium (HWE); Repeated articles, commentaries, editorials, case reports, book chapters, reviews, and research with insufficient data are all excluded. The application of these standards ultimately leads to 5 studies that meets the conditions for meta-analysis.

Information sources: The databases of PubMed, EMbase, Cochrane Library, Chinese National Knowledge Infrastructure (CNKI), Wanfang Database, and VIP Database.

Main outcome(s): This meta-analysis shows that the VDR FokI locus increased the risk of sepsis in patients. And it can provide a direction for the study of VDR gene polymorphism and sepsis, and provide a favorable research perspective on the prevention and treatment of sepsis from the perspective of gene polymorphism.

Additional outcome(s): VDR ApaI, BsmI, TaqI, and FokI gene polymorphism with the risk of sepsis.

Data management: Table of Excel and Forest plot are used for data aggregation and classication, while Review Manager 5.4 is used for data statistics and analysis.

Quality assessment / Risk of bias analysis: The effectiveness of inclusion in the study was evaluated separately by two reviewers (LQ and CML). Case-control studies were evaluated using the Newcastle Ottawa scale (NOS), with scores ranging from 0 to 4, 5 to 7, and 8 to 10 representing low, medium, and high quality, respectively. Finally, for statistical analysis, we selected a case-control study with a NOS score of 6.

Strategy of data synthesis: The statistical tests were performed using Review Manager 5.4. The following data was extracted by two authors from the included studies. The name of the lead author and

Li et al. Inplasy protocol 202340045. doi:10.37766/inplasy2023.4.0045 Downloaded from https://inplasy.com/inplasy-2023-4-0045/

the year of publication of the study; Country; The basic characteristics of the subjects, such as research category, sample size, average age or age range, genotype, total sample size, and case and control groups with US Chinese genotypes; Taking VDR Apa-I as an example, the required data were extracted according to allele model (A vs a), dominant model (AA vs Aa+aa), recessive model (AA+Aa vs aa), Codominance model homozygote model (AA vs aa), and Overdominance model (AA+aa vs Aa). We followed the procedure outlined in other studies to convert the Spearman correlation coefficient to Pearson correlation coefficient for inclusion in this article.

Subgroup analysis: For the correlation of VDR gene polymorphism with sepsis, we performed a subgroup analysis by dividing the study into Asia, Europe and Africa, according to the continent where the article study was completed.

Sensitivity analysis: We removed each study one at a time, recalculated the pooled P or OR estimates for the remaining studies, converted the fixed effects model and random effects model to each other to observe the stability of the results, and calculated the contribution of each included study to the pooled estimate in order to assess the sensitivity analysis.
When eliminating one research at a time or changing the random effects model to the fixed effects model for Fok I VDR gene polymorphism with sepsis, their data did not significantly alter the estimation of the pooled points. Thus, our results are quite trustworthy

Country(ies) involved: China (Guizhou University of Traditional Chinese Medicine).

Keywords: meta-analysis, vitamin D receptor, polymorphism, sepsis.

Contributions of each author:
Author 1 - Qian Li - Conceived of the study, participated in the design, collected the data, performed statistical analyses, Writing-original draft, and Data curation.
Email: qian_li1205@163.com
Author 2 - Menglu Chen - Article revision/review.
Email: 630773699@qq.com
Author 3 - Wen Li - Article revision/review, Data curation.
Email: aladdin64@163.com
Author 4 - Yihui Chai - Investigation.
Email: 846956178@qq.com
Author 5 - Liancheng Guan - Data curation.
Email: 404826084@qq.com
Author 6 - Yunzhi Chen - Project administration/Funding acquisition.
Email: chenyunzhi270@gzy.edu.cn

Li et al. Inplasy protocol 202340045. doi:10.37766/inplasy2023.4.0045 Downloaded from https://inplasy.com/inplasy-2023-4-0045/
